# Intravitreal Administration of Adalimumab‐Loaded Poly(Lactic‐co‐Glycolic Acid) Nanoparticles: Effects on Biodistribution and Pharmacokinetics

**DOI:** 10.1002/smsc.202400494

**Published:** 2025-01-27

**Authors:** Xurxo García‐Otero, Rubén Varela‐Fernández, Andrea Cuartero‐Martínez, Noemí Gómez‐Lado, Miguel González‐Barcia, Cristina Mondelo‐García, Carolina Feitosa, Pablo Aguiar, Anxo Fernández‐Ferreiro, Francisco J. Otero‐Espinar

**Affiliations:** ^1^ Department of Pharmacology Pharmacy and Pharmaceutical Technology Faculty of Pharmacy University of Santiago de Compostela (USC) 15782 Santiago de Compostela Spain; ^2^ Molecular Imaging Biomarkers and Theragnosis Lab Center for Research in Molecular Medicine and Chronic Diseases (CiMUS) University of Santiago de Compostela (USC) 15782 Santiago de Compostela Spain; ^3^ Nuclear Medicine Service and Molecular Imaging Group Health Research Institute of Santiago de Compostela (IDIS) 15706 Santiago de Compostela Spain; ^4^ Paraquasil Group Health Research Institute of Santiago de Compostela (IDIS) 15706 Santiago de Compostela Spain; ^5^ FarmaChusLab Group Health Research Institute of Santiago de Compostela (IDIS) 15706 Santiago de Compostela Spain; ^6^ Hospital Pharmacy Department University Hospital Complex of Santiago de Compostela (CHUS) Travesía da Choupana s/n 15706 Santiago de Compostela Spain; ^7^ Institute of Materials (iMATUS) University of Santiago de Compostela (USC) 15782 Santiago de Compostela Spain

**Keywords:** adalimumab, controlled releases, in vivo distribution and pharmacokinetics, nanoparticles, poly(lactic‐co‐glycolic acid)

## Abstract

Adalimumab, a monoclonal antibody used for treating inflammatory diseases, including eye diseases, faces challenges in biodistribution and targeted delivery. Nanoparticle (NP)‐based drug delivery systems have shown promise in enhancing the pharmacokinetic profiles of biologic drugs. This study aims to develop, and characterize intravitreal adalimumab‐loaded poly(lactic‐co‐glycolic acid) (PLGA) NPs to improve antibody distribution and therapeutic efficacy. Characterization studies, morphological examination, and quantitative, stability, and physical properties are conducted. In vitro release kinetics are assessed using a dialysis membrane method. In vivo biodistribution is studied in rats after intravitreal administration by Positron Emission Tomography/Computed Tomography imaging. The optimized NPs were spherical (around 300 nm) with a surface charge of about −20 mV. Encapsulation efficiency and drug loading reach values close to 100%. Stability studies showed minimal changes in particle size and drug content. In vitro release showed a biphasic pattern with an initial burst release followed by sustained release. Safety studies indicated no significant cytotoxicity or adverse effects. The adalimumab‐loaded PLGA NPs demonstrate favorable physicochemical characteristics, stability, and release profiles. In vivo distribution revealed a change in the antibody's distribution pattern after intravitreal administration via NPs encapsulation. These findings suggest the potential for enhanced therapeutic outcomes and warrant further investigation in disease‐specific models to explore the clinical potential of this NP‐based delivery system.

## Graphical abstract

Adalimumab‐loaded poly(lactic*‐co*‐glycolic acid) nanoparticles are developed to improve biodistribution and targeted delivery for inflammatory eye diseases. The system demonstrates favorable physicochemical properties, high encapsulation efficiency, sustained release, and minimal cytotoxicity. In vivo studies suggest modified biodistribution with potential therapeutic benefits. This approach highlights promising advancements in biologic drug delivery, warranting further exploration in disease‐specific models.

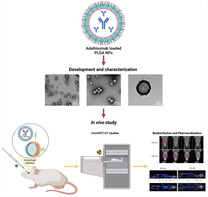

## 1 Introduction

In the realm of pharmaceutical research, the design, development, and characterization of drug delivery systems have garnered significant attention due to their potential to enhance therapeutic efficacy and reduce adverse effects of therapeutic agents. Controlled and modified release systems play a pivotal role in achieving these objectives by providing sustained drug release profiles, improving patient compliance, and minimizing dosing frequency.^[^ ^1^ ^]^

Controlled release systems are designed to release the drug at a predetermined rate over an extended period, maintaining therapeutic concentrations within the desired range.^[^ ^2^ ^]^ Modified release systems, in contrast, alter the drug release kinetics to optimize its pharmacokinetic profile, thereby improving efficacy and reducing side effects. These systems offer advantages such as reduced dosing frequency, enhanced patient adherence, and minimized fluctuations in drug plasma levels,^[^ ^3^ ^]^ among others.

Nanoparticles (NPs), with their unique properties such as high surface area‐to‐volume ratio, tunable size, and surface characteristics, present themselves as versatile systems for encapsulating and delivering a wide range of therapeutic agents.^[^ ^4^ ^]^ Their ability to protect drugs from degradation, target specific tissues, and control drug release kinetics makes them attractive for various applications in drug delivery.^[^ ^5^ ^]^ NPs can be tailored to encapsulate both hydrophilic and hydrophobic drugs, allowing for versatile delivery strategies.^[^ ^6^ ^]^

Monoclonal antibodies represent a class of biopharmaceuticals that have revolutionized the treatment of various diseases by targeting specific antigens with high specificity and affinity. These antibodies can modulate immune responses, inhibit cell signaling pathways, and neutralize pathogenic targets, making them valuable tools in precision medicine and targeted therapy.^[^ ^7^ ^]^ Adalimumab, a monoclonal antibody targeting tumor necrosis factor alpha (TNF‐*α*), has demonstrated remarkable efficacy in the management of autoimmune disorders such as rheumatoid arthritis, psoriasis, and inflammatory bowel disease. Its ability to reduce inflammation and modulate immune responses has positioned it as a cornerstone therapy for various chronic inflammatory conditions.

Adalimumab's use in ophthalmology has changed the way noninfectious uveitis is treated, especially in patients that don't respond to traditional immunosuppressive treatments. It has demonstrated potential in managing ocular inflammation, lowering the need for corticosteroids, and maybe enhancing long‐term visual results due to its capacity to neutralize TNF‐*α*. Adalimumab has also shown promise in the treatment of various inflammatory problems affecting the eyes, including as scleritis, orbital inflammatory disease, and abnormalities of the ocular surface linked to systemic autoimmune diseases. Adalimumab is administered either subcutaneously or intravenously, which poses challenges in terms of ocular bioavailability due to the blood–retinal barrier. This has led to consider alternative routes of administration, such as intravitreal, to improve the efficacy of treatment in ocular pathologies. Intravitreal administration of adalimumab offers the advantage of direct delivery to the site of action, potentially increasing efficacy and reducing systemic side effects. However, this route of administration is not routinely used in the treatment of uveitis or other ocular pathologies such as age‐related macular degeneration. Although there are case reports describing intravitreal use of adalimumab with empiric guidelines, no standardized or approved dosing regimen has been established for this route of administration. Intravitreal use of adalimumab is currently considered off‐label, underscoring the need for rigorous clinical studies to determine its safety and efficacy.

The integration of adalimumab into poly(lactic‐co‐glycolic acid) (PLGA) NPs presents a promising strategy to enhance its therapeutic potential through improved stability, prolonged circulation time, and targeted delivery to specific sites of inflammation.^[^ ^8^ ^]^ The implementation of delayed‐release systems for intravitreal adalimumab could revolutionize the treatment of inflammatory ocular pathologies. This approach would not only facilitate the arrival of the antibody to the site of action but would also extend the interval between required injections, potentially improving treatment efficacy and the quality of life of patients. This article aims to delve into the design, development, and characterization of adalimumab‐loaded PLGA NPs, highlighting their potential applications in the realm of controlled drug delivery and personalized medicine.

## 2 Materials

Resomer RG 503 H (Mw: 38 000 Da; lactide:glycolide = 50:50) was purchased from Evonik (Essen, Germany). Adalimumab was used as the Imraldi commercial formulation, which was provided by the Pharmacy Service of the University Clinical Hospital of Santiago de Compostela (Galicia, Spain). Polyvinyl alcohol (PVA) was purchased from Sigma‐Aldrich (St Louis, USA). Poloxamer 407 (Pluronic F 127) was acquired from Sigma‐Aldrich (St Louis, USA). Dichloromethane was purchased from Labkem Labware SL (Vilassar de Dalt, Barcelona). All other chemical reagents were of analytical grade.

## 3 Methods

### 3.1 Preparation of Adalimumab‐Loaded PLGA NPs

The preparation of the adalimumab‐loaded PLGA NPs was performed by a modified W/O/W double‐emulsion/evaporation method, as previously described by Sousa et al.^[^ ^9^ ^]^ with minor modifications. Two different types of NPs were prepared, one with and the other without a gelled core (hereinafter labeled as ADA PLGA NPs and ADA–GC PLGA NPs, respectively). For the preparation of the ADA PLGA NPs, a first emulsion was made by combining an organic phase, composed of 1% w/v PLGA in dichloromethane, and an aqueous phase, formed by a 2% w/v PVA solution containing the antibody, in a 1:3 aqueous:organic phase ratio. The emulsification process was carried out by sonication (Bandelin Sonoplus HD3200, Germany) at 70% amplitude for 30 s. The second emulsion was formed by including the first emulsion in a second aqueous phase consisting of 2% w/v PVA, in a 1:5 phase ratio, under the same sonication conditions. Once the W/O/W double emulsion was obtained, it was placed in a beaker under magnetic agitation (Heidolph Promax 2020, Germany) at 300 rpm for 3 h to promote the complete evaporation of the organic solvent. Hereafter, the resulting NPs were washed three times, after alternating centrifugation cycles (14 000 rpm, 4 °C, 90 min). Finally, the PLGA NPs were reconstituted in Milli‐Q water, the cryoprotectant (trehalose) was added and NPs were finally lyophilized (LyoQuest‐85 Telstar, Spain).

To point out, the ADA–GC PLGA NPs were prepared following the same preparation procedure but changing the original aqueous phase of the first emulsion by a 10% w/v Poloxamer 407 aqueous solution containing the antibody. Blank PLGA‐based NPs were prepared following the same experimental procedures previously described, but without adding the antibody to the first emulsion (hereinafter labeled as Blank PLGA NPs and Blank‐GC PLGA NPs).

### 3.2 Physicochemical Characterization of PLGA NPs

#### 3.2.1 Particle Size, Size Distribution, and *ζ* Potential

The physicochemical characterization of the adalimumab‐loaded PLGA NPs was performed as previously described by Varela et al.^[^ ^10^ ^]^ Briefly, a dynamic light scattering (DLS) system (Malvern Zetasizer Nano ZS instrument [ZEN3600, Malvern Instruments Ltd., Malvern, UK]) was employed to determine particle size, size distribution, and *ζ* potential values. Samples were previously diluted 1:10 in Milli‐Q water, and subsequently placed in nonrefundable cuvettes (DTS1070, Malvern Instruments Ltd., UK) for the analysis, at room temperature. DLS subsets were defined as previously detailed.^[^ ^10^ ^]^ Each sample was analyzed in triplicate.

#### 3.2.2 Morphological Evaluation

PLGA‐based NPs were assessed by scanning electron microscopy (SEM) and transmission electron microscopy (TEM) analysis. Formulations were placed on a metal stub double‐sided conductive adhesive tape and subsequently iridium sputter‐coated prior to the SEM analysis (ZEISS EVO LS 15/EDX, ZEISS) (Jena, Germany), under different magnifications. In contrast, samples were stained with 2% w/v phosphotungstic acid for 10 min, placed on copper grids with Formvar film and dried overnight for TEM analysis by using a JEOL JEM‐F200CF‐HR microscope (JEOL) (Peabody, USA). The air‐dried samples were imaged using a predefined acceleration voltage.

#### 3.2.3 Production Yield

The production yield (PY) of the adalimumab‐loaded PLGA NPs was assessed as described in previous works.^[^ ^10^ ^]^ In brief, 1.5 mL of final formulations was centrifuged at 14 000 rpm and 4 °C for 90 min to promote the NPs sedimentation. Once centrifuged, the supernatant was removed, and sediment was vacuum‐dried until constant weight. The resulting data for PY was finally obtained by applying the following mathematical equation

(1) $$\text{Production yield }\left(\text{PY}\right)=\frac{\text{Nanoparticles weight}}{\text{Total initial solids weight}}\times 100$$

#### 3.2.4 Encapsulation Efficiency and Loading Capacity of NPs

The assessment of the adalimumab‐loaded PLGA NPs in terms of encapsulation efficiency (EE) and loading capacity (LC) was performed after isolation by centrifugation, as previously described.^[^ ^10^ ^]^ The amount of unentrapped antibody was determined in the supernatant by ultra‐performance liquid chromatography (UPLC). The UPLC system (ACQUITY UPLC H‐Class PLUS Bio) was equipped with a solvent delivery quaternary pump, a two‐rack autosampler manager, column heaters, multicolumn managers, a Diode Array Detector HS, and 15 000 bar maximum pressure.

The analysis was performed under a gradient method (see gradient method details in **Table** **1**, using two different mobile phases, these being: 1) Phase A, composed of a 0.1% v v^−1^ trifluoroacetic acid (TFA) acetonitrile solution, and 2) Phase B, composed of a 0.1% v v^−1^ TFA aqueous solution. A seal wash solution composed of a 0.1% v v^−1^ TFA acetonitrile/water (40:60 v v^−1^) solution was employed as a rinsing medium between each injection.

**Table 1:** Specifications of the UPLC gradient method for the adalimumab quantification.

Time [min]	Flow [mL min^−1^]	Phase A [%]	Phase B [%]
0.00	0.8	15	85
2.50	0.8	45	55
2.51	0.8	35	65
5.00	0.8	35	65
5.50	0.8	15	85

A 1 μL injection volume and a 2.3 min retention time were previously settled. A BioResolve RP mAb column (polyphenyl, 450 Å, 2.7 μm, 2.1 × 50 mm, 1/pk) was used as the separation medium, at an 80 °C prefixed temperature. A 280 nm wavelength was employed for the adalimumab quantification. Data processing was performed by means of the Empower software. The analytical method was previously validated according to International Conference on Harmonization (ICH) guidelines,^[^ ^11^ ^]^ and mathematical adjustments were subsequently applied by means of the GraphPad Prism software (GraphPad Software 8 v.8.2.1, San Diego, CA, USA, 2019).

Resulting data were obtained by applying the following mathematical equations

(2) $$\text{EE}=\;\frac{\text{Total amount of antibody}-\text{Amount of unentrapped antibody}}{\text{Total amount of antibody}}\times 100$$

(3) $$\text{LC}=\;\frac{\text{Total amount of antibody}-\text{Amount of unentrapped antibody}}{\text{Nanoparticles weight}}\times 100$$

#### 3.2.5 Effect of Antibody Amount on the NP's Physicochemical Properties

The effect of the amount of adalimumab on the physicochemical properties of the formulations was evaluated in terms of size, size distribution, and surface charge. These data were then correlated with the values of encapsulation efficiency and loading capacity. Each formulation was measured in triplicate.

### 3.3 Stability Studies

#### 3.3.1 Stability to Storage

The stability to storage assessment of the adalimumab‐loaded PLGA NPs was performed following the same procedure detailed in previous works,^[^ ^10^ ^]^ by subjecting the particles to different temperature values: 1) 4 ± 2 °C, 2) 25 ± 2 °C, and 3) 37 ± 2 °C for a 3 month period. A DLS system (Zetasizer Nano, Malvern Instruments Ltd.) (Worcestershire, UK) was furtherly employed in the assessment of the particle size, size distribution, and surface charge, carrying out each measurement in triplicate.

#### 3.3.2 Stability to pH

The pH conditions of the media were one of the main key factors affecting the stability of colloidal systems. The stability to pH of the adalimumab‐loaded PLGA NPs was studied along the entire pH interval (from pH 2 to 12), as detailed in previous works.^[^ ^10^ ^]^ A 500 mL volume of each sample was included into a final 5 mL volume of double‐distilled water for 24 h at 4 ± 2 °C, prior to measurement. A DLS system (Zetasizer Nano, Malvern Instruments Ltd.) (Worcestershire, UK) was furtherly employed in the assessment of the particle size, size distribution, and surface charge, carrying out each measurement in triplicate.

#### 3.3.3 Stability to Ionic Strength

The stability to ionic strength of the adalimumab‐loaded PLGA NPs was studied along a predefined ionic strength interval (from 0.2 to 2.0 m), as specified in preceding studies.^[^ ^10^ ^]^ A 500 mL volume of each sample was included into a final 5 mL volume of double‐distilled water and then incubated at 4 ± 2 °C for 24 h, prior to measurement. A DLS system (Zetasizer Nano, Malvern Instruments Ltd.) (Worcestershire, UK) was furtherly employed in the assessment of the particle size, size distribution, and surface charge, carrying out each measurement in triplicate.

### 3.4 Differential Scanning Calorimetry and Thermogravimetry Analysis

Differential scanning calorimetry (DSC) is one of the most commonly used techniques in powder characterization for thermal analysis of different processes.^[^ ^12^, ^13^ ^]^ Changes in solid structure and interactions between the antibody and the polymer may be studied by this technique, which was carried out by means of a TA Instruments Q1000 DSC/thermogravimetry analysis (TGA)/infrared (IR) analyzer (Madrid, Spain), calibrated with indium. DSC curves were acquired by a 10 °C min^−1^ scanning rate through a predefined temperature range (from 0 to 150 °C at a 10 °C min^−1^ heating rate), in a nitrogen environment (50 mL min^−1^ flow rate). Each formulation was tested in triplicate. Likewise, TGA analysis is one of the main useful methods for the determination of the presence or absence of residual solvents in formulations prepared by the W/O/W double‐emulsion/solvent evaporation method. The procedure's schedule follows the same pattern described for the DSC analysis. Each formulation was also tested in triplicate.

### 3.5 Fourier‐Transformed Infrared Spectroscopy Analysis

Fourier‐transformed infrared (FTIR) spectroscopy analysis is frequently used to identify the molecule structures with IR‐radiation absorption characteristics according to their molecular vibration. FTIR assay performed to structurally characterized the adalimumab‐loaded PLGA NPs. Certainly, samples were scanned through the IR interval (from 400 to 4000 cm^−1^; 4 cm^−1^ resolution and dry air as a background blank) (GladiATR, Varian Pike Technologies). The spectra of each sample were recorded at a 64 scans min^−1^ speed, and resulting data were processed by means of the Resolutions Pro software. Each formulation was tested in triplicate.

### 3.6 X‐Ray Diffractometry Analysis

X‐ray diffraction is one of the most widely used techniques in the study of solid materials. At present, this technique is moving toward the analysis of structured samples in submicron dimensions and toward the structural analysis of nonsolid systems. The X‐ray diffractometry study was carried out following the ISO 9001:2015 standard normative in terms of reception, sample management, and analysis. All samples were preserved at room temperature prior to the analysis.

Adalimumab‐loaded PLGA NPs structure was analyzed by wide‐angle X‐ray (WAX) diffraction by using an X‐ray diffractometer (Philips X’Pert, USA), operated with a PW170 control unit, a PW1820/00 vertical goniometer and an Enraf Nonius FR590 generator. Samples were scanned from a Cu‐ *K*α source, monochromated with a graphite monochromator (λ = 1.5406 Å), at 40 kV and 30 mA, using 2*θ* from 2° to 50° at a scan rate of 0.04° min^−1^. Samples were rotated during the analysis to obtain the most optimal peak profiles for the diffractograms, as well as to minimize the effect of the preferred orientation. It also must be taken into account that formulations were deposited in oriented glass bases to avoid background. The mathematical analysis of the diffractograms was performed by means of the HighScore Plus 3.0d software.

### 3.7 In Vitro Release Study

The adalimumab release and kinetic behavior from PLGA NPs was established by a dialysis approach in a physiological‐like environment. As such, freshly prepared adalimumab‐loaded PLGA NPs were thrice washed and resuspended in a suitable aqueous solution (pH 7.4 and 0.075 ionic strength) for a further proof of concept.^[^ ^14^ ^]^ The antibody release rate from the PLGA NPs was finally determined at predetermined times by UPLC, following the exact same procedure employed for the EE and LC determination (see details in Section 3.2.4.), at a 280.0 nm wavelength. Data processing was carried out as cumulative release data and adjusted for available diffusion surface. Each formulation was tested in triplicate.

### 3.8 Toxicity Analysis

#### 3.8.1 Bovine Corneal Opacity and Permeability

The bovine corneal opacity and permeability (BCOP) test is a commonly utilized organotypic model for evaluating formulations with potential ocular irritancy to adhere to animal replacement principles.^[^ ^15^ ^]^ The foundational principles of the BCOP test were initially established by Tchao et al. and refined by Gautheron et al.^[^ ^16^, ^17^, ^18^ ^]^ Corneal opacity changes were assessed using luxometry and UV–vis spectrophotometry techniques, while permeability data was specifically measured at 490.0 nm wavelength using UV–vis spectrophotometry.

BCOP has been developed mainly to study the toxicity of systems administered topically on the surface of the eye. However, it may be of interest to study the potential toxicity of intravitreal formulations that may distribute and accumulate in the anterior chamber, as it is described for large molecules, such as monoclonal antibodies.

The methodology of this assay was previously detailed,^[^ ^10^ ^]^ where bovine corneas were exposed to the NPs in the BCOP test chambers, and changes in opacity and permeability were monitored. Data on corneal health effects were collected, analyzed, and compared with control groups to assess cytotoxicity. Each formulation underwent triplicate evaluations, and the resulting opacity and permeability values were utilized to calculate an in vitro score value, as outlined in the following equation

(4) $$\text{IVIS}=\text{mean opacity value}+\left(15\times {\text{mean permeability OD}}_{490}\text{ value}\right)$$

#### 3.8.2 Hen's Egg Test on the Chorioallantoic Membrane

A useful organotypic model for evaluating possible irritants in vitro for ophthalmological applications is the hen's egg test on the chorioallantoic membrane (HET–CAM) test. By using the CAM of fertilized chicken eggs as a stand‐in for ocular tissues, this technique replaces conventional in vivo irritation testing with an affordable and morally acceptable option. The HET–CAM test fundamentals are based on the determination of acute ocular irritation or corrosion,^[^ ^19^ ^]^ by assessing the occurrence or nonoccurrence of adverse effects, mainly hemorrhage, lysis, or coagulation along the CAM. An irritation score was further calculated following the criteria of Kalweit et al. if needed.^[^ ^20^ ^]^

The usefulness of HET–CAM lies on its ability to imitate the vascular reactions of the conjunctiva and retina to potentially irritating agents. In weight of evidence and tiered testing methodologies, the HET–CAM assay is still a useful tool for evaluating the safety of possible irritants in ocular and mucosal applications. The methodology of this assay was previously detailed,^[^ ^10^ ^]^ where formulations were applied onto the CAM of viable fertilized eggs at 9th day after incubation. A 5 min observation period was staged, where images were taken prior and after the assay, and data was further analyzed, both individually and in combination. Each sample was tested in triplicate.

### 3.9 Biological Activity of Adalimumab‐Loaded PLGA NPs

The determination of the biological activity of adalimumab from the adalimumab‐loaded PLGA NPs was performed using a commercial enzyme‐linked immunosorbent assay (ELISA) kit (Promonitor‐ADL, Proteomika S.L., subsidiary of Progenika Biopharma S.A., Spain) and a Triturus ELISA Instrument (Grifols, Barcelona, Spain). Specifically, an ELISA with wells precoated with TNF‐*α* was employed.

This ELISA employs a solid substrate coated with TNF‐*α* to capture the adalimumab released from the PLGA NPs. Briefly, adalimumab was released from the PLGA NPs following the method previously described (see details in Section 3.2.4.) and supernatant was then added to the TNF‐*α*‐coated microwells, allowing the antibody to form complexes with the immobilized TNF‐*α*. After incubation and washing steps, a horseradish peroxidase (HRP)‐conjugated monoclonal antibody specific for the adalimumab portion of the adalimumab/TNF‐*α* complexes was added. Following another incubation and washing cycle, a chromogenic substrate, 3,3′,5,5′‐tetramethylbenzidine (TMB), was introduced. The enzymatic reaction catalyzed by the HRP resulted in a color change, with its intensity proportional to the amount of adalimumab in the sample.

The absorbance of the colored product was measured by spectrophotometry using the Triturus ELISA Instrument, and adalimumab concentrations were determined by comparing the sample values to the standards provided in the kit, enabling quantification of adalimumab activity in the analyzed samples. Each sample was tested in triplicate.

### 3.10 Positron Emission Tomography Biodistribution and Pharmacokinetics of Intravitreal Adalimumab‐Loaded PLGA NPs

A methodology pioneered by our group in previous studies^[^ ^21^, ^22^ ^]^ was employed to investigate pharmacokinetics and biodistribution using positron emission tomography/computed tomography (PET/CT) imaging. For this purpose, adalimumab was conjugated and radiolabeled, then intravitreally administered into rats, and subsequently, whole‐body biodistribution and pharmacokinetics was derived from PET/CT image analysis.

#### 3.10.1 Antibody Conjugation

The antibody conjugation process is the first step for the study of its pharmacokinetics by radio‐imaging techniques. In the present work, conjugation was performed following the protocol previously described by Verel et al.^[^ ^23^ ^]^ with slight modifications. Briefly, an adalimumab commercial formulation (Imraldi, Samsung Bioepis NL) was purified to obtain pure adalimumab, by means of Amicon Ultra centrifugal tubes (30 KDa NMWL, Merck Millipore), using Milli‐Q water as a rinsing medium.

Once purified, the adalimumab conjugation process was initiated; for this purpose, tetrafluorphenil‐N‐succinyldesferrioxamine‐B‐Fe^3+^ (TFP‐N‐sucDf‐Fe) (referred as DFO) was used as a conjugation agent, zirconium being used as the complementary radiotracer. The conjugation was carried out at room temperature, where DFO was added in double excess molar to the amount of antibody. Both components were incubated for 30 min at a pH 9.5–10, previously adjusted by using a 1.0% w v^−1^ Na_2_CO_3_ aqueous solution. After conjugation, the pH of the resulting solution was adjusted to 4.0–4.5 by using a 2.5% w v^−1^ H_2_SO_4_ aqueous solution. A 50‐fold molar excess of a 2.5% w v^−1^ ethylenediaminetetraacetic acid (EDTA) aqueous solution was then added and solution was re‐incubated at 35 °C for 30 min. Conjugated adalimumab was repurified and finally stored at −80 °C until NPs preparation.

To point out, the conjugated adalimumab‐loaded PLGA‐based NPs were obtained by the same procedure described in previous sections (see details in Section 3.1.) but replacing adalimumab by the conjugated adalimumab.

#### 3.10.2 Labeling of the Adalimumab‐Loaded PLGA NPs

Adalimumab labeling procedure was carried out using an incubation method,^[^ ^23^ ^]^ where ^89^Zr was used as the labeling radiotracer, certainly as an ^89^Zr–oxalate acidic aqueous solution (9.0% w v^−1^ oxalic acid) (BV Cyclotron VU, PerkinElmer). The pH of the radiotracer solution was first adjusted to a 4.0–4.5 value by using a 2.0% w v^−1^ Na_2_CO_3_ aqueous solution and then included into a 4‐(2‐hydroxyethyl)‐1‐piperazineethanesulfonic acid buffered solution (referred as HEPES buffer; pH 7).

Once the labeling medium was prepared, conjugated adalimumab‐loaded PLGA‐based NPs were added, and resulting suspension was incubated at room temperature for a 90 min period. The radiotracer activity was prefixed prior to the incubation procedure, adjusted to the NP's concentration for the distribution and pharmacokinetic analysis.

A proof‐of‐concept test was carried out prior to the labeling of the NPs to observe the behavior of the radiotracer. For this purpose, blank NPs and antibody‐loaded NPs were freshly prepared, and the radiotracer (5.55 MBq) was then added to the NPs and incubated for 90 min at room temperature. After this period, the NPs were centrifuged (18 000 rpm for 30 min at 25 °C) and the supernatant was separated from the sediment. The activity of both supernatant and sediment was subsequently measured, and a comparison was made between the two groups.

#### 3.10.3 Experimental In vivo Evaluation of Adalimumab‐Loaded PLGA NPs Pharmacokinetics after Intravitreal Administration

The in vivo pharmacokinetics study of the adalimumab‐loaded PLGA NPs was carried out at the Center for Experimental Biomedicine of the University of Santiago de Compostela (referred as CEBEGA). The study was performed on male Sprague Dawley rats (≈350 g weight), which were kept under the optimal care and maintenance conditions established by the research center until the study: a temperature of 22 ± 1 °C, humidity at 60 ± 5%, and a 12 h light/dark cycle regulated by artificial lighting. The animals had unlimited access to food and water. All procedures involving the animals adhered to the association for research in vision and ophthalmology guidelines for the use of animals in ophthalmic and vision research, as well as the approved standards for laboratory animal care.^[^ ^24^ ^]^ The experiments received approval from the Committee for Ethical Research of the Health Research Institute of Santiago de Compostela (IDIS) (15012/2021/001) and were conducted in compliance with Spanish and European Union regulations (86/609/CEE, 2003/65/CE, 2010/63/EU, RD 1201/2005, and RD 53/2013).

For this study, healthy rats (*n* = 2, 4 eyes) were intravitreally injected with encapsulated ^89^Zr–radiolabeled ADA–GC NPs.

##### Intravitreal Administration of Adalimumab‐Loaded PLGA NPs

The rats were placed in a veterinary gas chamber filled with a 3% v v^−1^ isoflurane/oxygen inhalation solution (Baxter, Deerfield, Illinois, USA). Once anesthetized, they were placed on a surgical table, maintaining anesthesia via a specific face mask for this animal species with a 2.5% v v^−1^ isoflurane/oxygen inhalation solution. Intravitreal injections were administered following the procedure outlined in our previous publications.^[^ ^21^, ^25^ ^]^ Both eyes received topical anesthetic eye drops (a mixture of 1 mg mL^−1^ tetracaine hydrochloride and 4 mg mL^−1^ oxybuprocaine hydrochloride) (Colircusí anestésico doble, Alcon Healthcare, Texas, USA) followed by mydriatic eye drops (10 mg mL^−1^ cyclopentolate hydrochloride) (Colircusí Ciclopléjico, Alcon Healthcare, TX, USA) to visualize the fundus prior to the injection procedure. The injection procedure was performed just before the study started, using a surgical microscope (Takagi OM‐5 220–2; Takagi, Tokyo, Japan). A 4 μL volume was injected into the vitreous through pars plana using a NanoFil syringe (WPI, Friedberg, Germany) fitted with a 35 G needle for each eye. An activity of 1.1–1.74 MBq of ^89^Zr–adalimumab was administered in each injection. Eyes with lens damage or significant bleeding during or after the intravitreal injection were discarded from the study.

##### PET Acquisition, Blood Sample Collection, and Analysis

A preclinical Albira PET/CT system (Bruker Biospin, Billerica, Massachusetts, USA) was used for PET imaging immediately following intravitreal administration. Two sequential acquisitions of 10 min per bed position were performed to capture the entire body of the rats. Throughout the acquisition, the animals were maintained under anesthesia with a 2.5% v v^−1^ isoflurane/oxygen inhalation solution via a face mask, continuously monitoring their respiratory rate. Scans were conducted at predefined time points (0, 2, 4, 8, 12, 24, and 36 h and daily from day 2 to 9) to obtain the pharmacokinetic profile. Single PET images were reconstructed using the maximum likelihood expectation maximization (MLEM) algorithm with 12 iterations and an image pixel size of 0.5 × 0.5 × 0.5 mm^3^. All images were analyzed using the Amide's Medical Image Data Analysis Tool software. Quantitative analysis involved manually drawing ellipsoidal regions of interest (ROIs) of 12 × 12 × 12 mm (904 mm^3^), significantly larger than the volume of the whole rat eye to encompass all radiotracer uptake, accounting for the commonly blurred contour of the radiotracer distribution due to the limited spatial resolution of PET scanners.

The mean radiotracer concentration from the initial image frame post‐intravitreal administration was used as the reference, with subsequent measurements from later frames expressed as a percentage of this initial uptake. Additional ROIs (7 × 7 × 7 mm, 343 mm^3^) were delineated for the liver, kidneys, and cervical lymph nodes activity quantification for each time point. The mean radiotracer concentration from these ROIs was adjusted for the radioactive decay of the ^89^Zr radioisotope (t_1/2_ = 3.3 days). The standardized uptake value (SUV) was calculated as the mean uptake of ^89^Zr–adalimumab, normalized by the injected activity and the body weight of the animal, according to the following equation:

(5) $$\text{SUV}=\frac{\text{Measured radioactivity concentration }\left(\text{Bq}/\text{mL}\right)\cdot \text{ Injected radioactivity }\left(\text{Bq}\right)}{\text{Body weight }\left(\text{Kg}\right)}$$

Finally, blood samples were obtained from the tail vein of each rat immediately after every PET acquisition, while anesthesia was maintained. Specifically, two aliquots of known volume (between 20 and 100 μL) were extracted and measured in a well counter (Atomlab Wipe Test Counter, Biodex, New York, USA). The activity was corrected for the radioactive decay of ^89^Zr. Total blood activity was calculated using the theoretical rat blood volume based on body weight, according to the following equation:^[^ ^26^ ^]^

(6) Blood volume=0.06×Body weight+0.77

**Figure 1:**
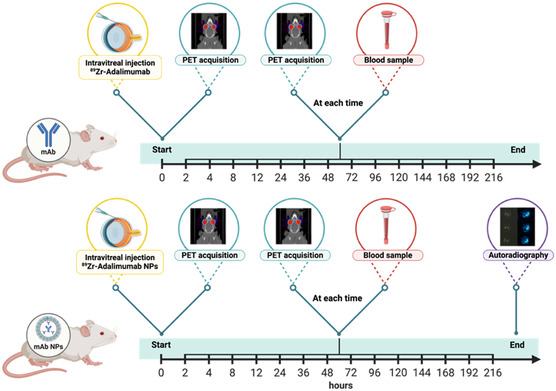
Study timeline for both groups (mAb and encapsulated mAb NPs group): intravitreal injections of ^89^Zr–adalimumab (data obtained from our previous work^[^ ^21^ ^]^) and encapsulated ^89^Zr–Adalimumab Gelled‐Core NPs (^89^Zr–ADA–GC NPs), PET acquisitions, blood sample collection, and autoradiography. Designed with BioRender.com.

Blood activity levels after intravitreal injection of ^89^Zr–adalimumab were reported as the ^89^Zr–adalimumab uptake value, normalized by the injected activity and the body weight of the animal (activity [%]). The experimental design of the study is illustrated in **Figure** **1**.

##### In Vivo Pharmacokinetic Analysis of Adalimumab‐Loaded PLGA NPs

The percentage of the remaining activity in the eye was fitted to the open two‐compartment models described in our previous work,^[^ ^21^ ^]^ whose equation model is

(7) $$X_{1}=\frac{D\left(\beta -k_{12}\right)}{\beta -\alpha }e^{-\alpha t}+\frac{D\left(k_{12}-\alpha \right)}{\alpha -\beta }e^{-\beta t}$$

where *X*_1_ represents the activity (expressed as % of remaining activity) in the eye compartment, *D* refers to the total administered activity, *a* and *b* are the hybrid constants of the model, and *k*_12_ is the micro‐constant of distribution between the eye (compartment 1), and the blood and well‐perfused organs (compartment 2). The hybrid constants can be expressed as

(8) $$\alpha +\beta =k_{12}+k_{21}+k_{20}$$

(9) $$\alpha \times \beta =k_{12}\cdot k_{21}$$

where *k*_21_ and *k*_20_ are the distribution micro‐constant from the compartment 2 to the eye and the elimination constant from compartment 2, respectively.

A one‐compartment model with first‐order absorption kinetics was used for the analysis of ^89^Zr–adalimumab distribution in blood and organs. Nonlinear fitting was performed with weighting schemes based on the predicted concentration. The best model was chosen based on the best fit, assessed by the calculated and observed concentration curve plots, the Akaike information criterion, and the lowest percentage of coefficient of variation (%) values.

##### Autoradiography

The animals, to which the ^89^Zr–labeled NPs were administered, were sacrificed 9 days after administration, once the PET study was finished. The animals were sacrificed by carbon dioxide inhalation and both eyes were enucleated. After this procedure, the preparation of the block including the sample for cutting in the cryostat was performed. For this purpose, the samples were placed in a mold containing tissue freezing medium (Leica Biosystems Nussloch GmbH, Nussloch, Germany) and placed into dry ice at −78.5 °C until they were completely frozen, allowing gradual freezing. The blocks were eventually stored at −20 °C until the subsequent slice and acquisition step in the autoradiography system.

A cryostat (Leica CM1520, Leica Biosystems Nussloch GmbH, Nussloch, Germany) was used to perform the sections of each sample. 20 μm sagittal slices of each eyeball were obtained and mounted on the anterior part of a SuperFrost Plus Adhesion Slides Microscope (Epredia, Breda, Netherlands). Autoradiography images were acquired employing a BeaQuant real‐time quantitative autoradiography system (Ai4r, Nantes, France) with a HEEL sample holder designed for energy photons greater than 150 KeV. For this purpose, a copper adhesive strip was placed on the back of the slide and the slides were subsequently left to dry at room temperature for at least 20 min. The 30 min acquisitions were performed for each sample. Images were analyzed using the Beamage v.3.5.2 and Beavacq v.2.1.6 software (Ai4r, Nantes, France).

#### 3.10.4 Statistical Analysis

All statistical analysis were conducted using GraphPad Prism version 9.5 (GraphPad Software, San Diego, CA, USA). Data were preprocessed by evaluating normality using the Shapiro–Wilk test, and outliers were identified and excluded based on the robust regression and outlier removal method with a *Q* value of 1%. Data transformations (e.g., logarithmic transformation) were applied to meet normality assumptions, when necessary.

Quantitative data are presented as mean ± standard deviation (SD) unless otherwise specified. Sample sizes for each experimental group are indicated in the figure legends. An unpaired Student's *t*‐test was employed for comparisons between two groups, while for experiments involving multiple groups, one‐way or two‐way analysis of variance (ANOVA) was performed, depending on the number of independent variables, followed by Tukey's post hoc test for multiple comparisons. All tests were two‐sided, and statistical significance was set at an alpha (*α*) level of 0.05. Non‐parametric alternatives, such as the Mann–Whitney *U* test or Kruskal–Wallis test, were used as appropriate for data that did not meet the assumptions of parametric tests.

For pharmacokinetic modeling, parameters such as half‐life, area under the curve, and clearance rate were calculated, and intergroup comparisons were conducted using ANOVA. Validity of model assumptions was verified before analysis. The *α* values are reported in the figures and text where relevant. An *α* value of less than 0.05 was considered statistically significant.

## 4 Results and Discussion

### 4.1 Preparation of Adalimumab‐Loaded PLGA NPs

Both types of adalimumab‐loaded PLGA NPs were successfully prepared by the modified W/O/W double‐emulsion/solvent evaporation method. This experimental procedure enabled the antibody encapsulation and stabilization in nanosized systems. A previous optimization process was carried out following a factorial study design, where critical parameters influencing the NP's preparation were evaluated.

### 4.2 Physicochemical Characterization of PLGA NPs

#### 4.2.1 Particle Size, Size Distribution, and *ζ* Potential

**Table** **2** shows the resulting data for the particle size, size distribution polydispersity index (PDI), and *ζ* potential for all the PLGA‐based formulations. As presented, all PLGA NPs showed a highly monodisperse and narrow distribution, evidencing those formulations were homogeneous and stable overtime, with no associated aggregation or caking phenomena. In addition, all formulations showed an adequate negative surface charge, possibly derived from the intrinsic charge of the polymer, allowing the suspension stability to be increased due to charge repulsion between the particles.

**Table 2:** Size, size distribution, and *ζ* potential of the PLGA‐based NPs.

Formulation	Drug:polymer ratio	Size [nm]	PDI	*ζ* Potential [mV]
Blank PLGA NPs	–	164.43 ± 5.26	0.062 ± 0.032	−19.30 ± 2.32
Blank‐GC PLGA NPs	–	274.18 ± 8.96	0.174 ± 0.037	−21.49 ± 2.50
ADA PLGA NPs	1:10	284.20 ± 7.31	0.081 ± 0.027	−18.87 ± 2.69
	1:25	283.80 ± 11.80	0.073 ± 0.029	−19.27 ± 1.88
	1:50	266.53 ± 8.13	0.077 ± 0.041	−18.72 ± 2.24
ADA–GC PLGA NPs	1:10	304.83 ± 24.47	0.177 ± 0.038	−17.26 ± 0.87
	1:25	271.89 ± 8.96	0.171 ± 0.037	−20.79 ± 2.65
	1:50	272.79 ± 7.87	0.157 ± 0.041	−19.02 ± 1.34

The *ζ* potential analysis is particularly noteworthy since it showed that the addition of the monoclonal antibody did not significantly change the surface charge of the NPs. The PLGA NPs’ *ζ* potential did not change either before or after adalimumab encapsulation. The little alteration in surface charge indicates that most of the adalimumab molecules are contained in the NPs’ core instead than being adsorbed onto their surface. This is an important insight for several reasons. First, adalimumab's internalization within the PLGA matrix suggests that the antibody is better shielded from putative biological milieu degradation agents. Second, it implies that a more regulated and prolonged release profile would be possible due to the release kinetics. The preservation of the initial surface characteristics of the PLGA NPs suggests that the addition of adalimumab does not considerably change the surface characteristics of the NPs.

#### 4.2.2 Morphological Evaluation

A morphological evaluation was performed in all PLGA‐based formulations by means of SEM and TEM analysis. Both types of NPs have an aqueous core, which is removed during the freeze‐drying process prior to the SEM analysis; because of this, the vast majority of NPs showed an amorphous appearance. It must be also considered that particle fusing, dimpling, or cracking phenomena may appear on several formulations, possibly associated with the sample preparation for SEM analysis (SEM images were not included).

**Figure 2:**
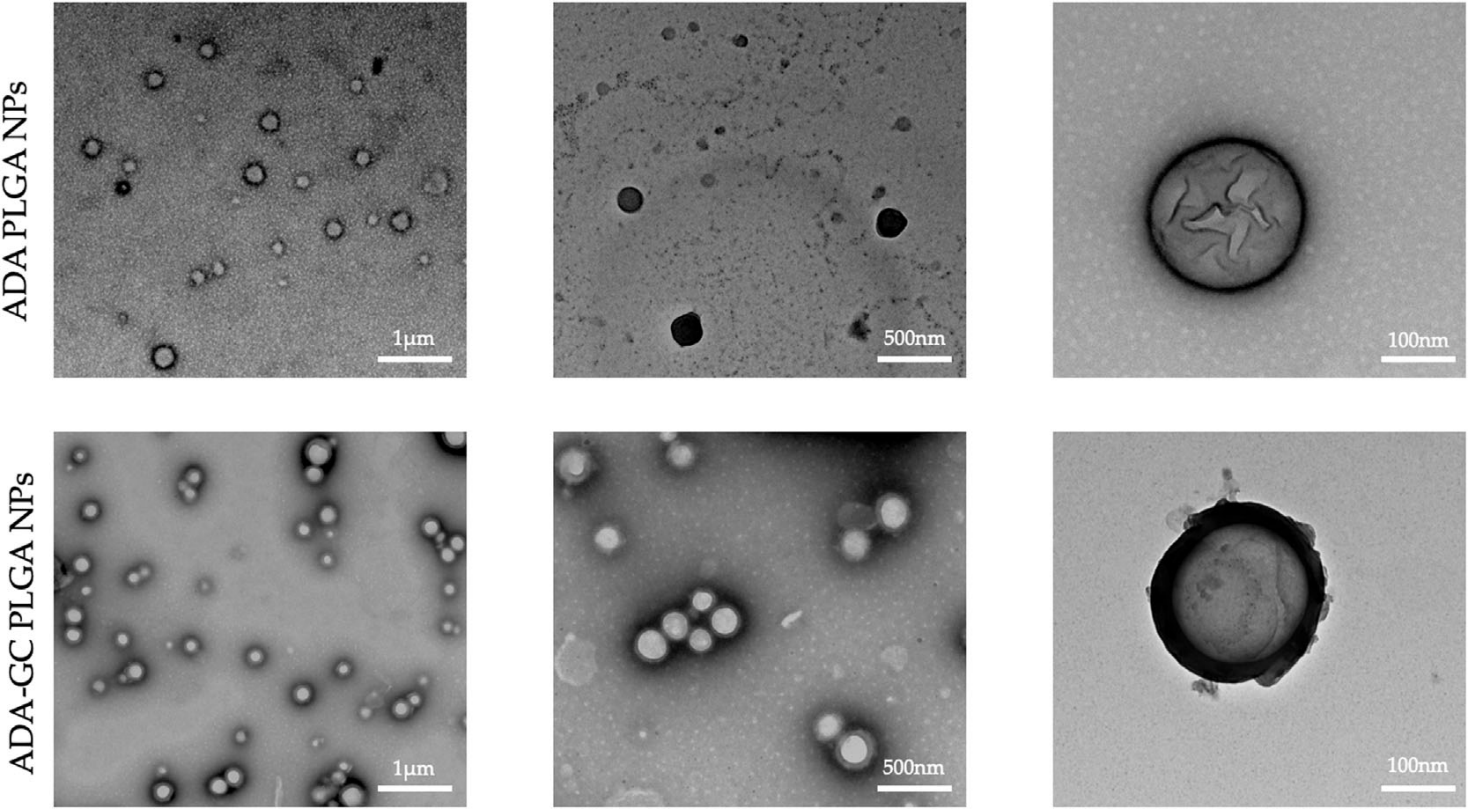
TEM images of adalimumab‐loaded PLGA nanoparticles.

Nevertheless, TEM analysis revealed that all NPs were round, uniform, and homogeneous, not fused, and had a smooth surface (see details in **Figure** **2**). In addition, this analysis confirmed that the NPs contained an internal aqueous core, thus obtaining a reservoir‐type polymeric system, a priori useful for controlled drug release processes.

#### 4.2.3 PY, EE, and LC

**Figure 3:**
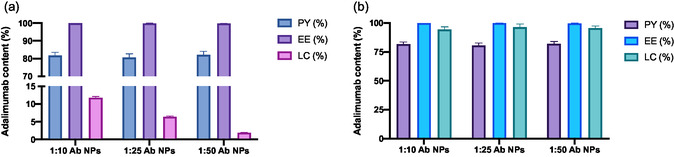
Resulting data of PY, EE, and LC of adalimumab‐loaded PLGA nanoparticles. a) Graph shows the quantitative data for the ADA PLGA NPs while b) the graph displays the results for the ADA–GC PLGA NPs.

The conventional W/O/W double‐emulsion procedure employed in the encapsulation of hydrophilic drugs (e.g., proteins, antibodies, etc.) into polymeric colloidal systems suffered from low EE and LC values, mainly due to the drug rapidly diffusion from the inner aqueous phase to the external aqueous phase, keeping appropriate PY values. Thus, a modified W/O/W double‐emulsion/solvent evaporation technique was used in the present work to improve the encapsulation of adalimumab into PLGA NPs. **Figure** **3** shows the PY, EE, and LC resulting data for all the PLGA‐based NPs. As observed, the NPs exhibited similar PY and EE values independent of the drug:polymer ratio. However, differences in the LC values were observed depending on the aforementioned ratio, where the increase is usually proportional to the amount of the drug added, suggesting that the NPs show sufficient capacity to encapsulate antibody in their core.

#### 4.2.4 Effect of Antibody Amount on the NP's Physicochemical Properties

**Figure 4:**
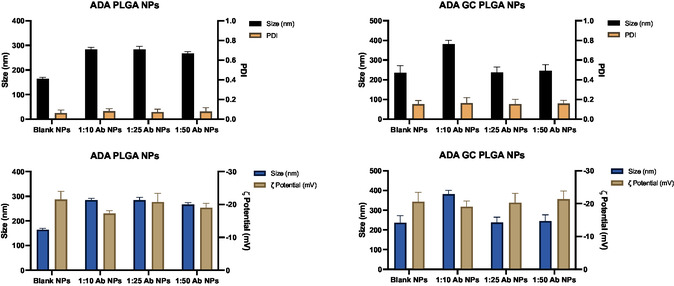
Size, size distribution, and *ζ* potential of the PLGA‐based NPs.

The effect of antibody concentration over the particle size, EE, and LC of PLGA NPs prepared by the double‐emulsion/evaporation technique is presented in **Figure** **4**. The effect of antibody amount on final particle size, size distribution, and surface charge may be described by the adalimumab influence on the droplet size of the inner phase of the first emulsion, which may modify its ability for dispersion in the outer aqueous phase. However, this effect was only limited and not significant in the case of particle size, while EE and LC values were significant critical parameters, affecting the PDI and *ζ* potential values.

The surface charge was also monitored for all the different formulations and its values ranged from ≈−15 to −25 mV for both types of formulations (see details in Table 2 of Section 4.2.1.). As observed, all *ζ* potential values were negative, being possibly attributed to the presence of carboxyl end groups of PLGA on the NPs external surface.

Likewise, based on the antibody amount, resulting data showed that EE were around 80% for both types of formulation (see details in Figure 3 of Section 4.2.3.), while LC values varied from 2.5 to 10% and 90 to 95% for ADA PLGA NPs and ADA–GC PLGA NPs, respectively, depending on the drug:polymer ratio.

Three different adalimumab concentrations were used in the NP preparations (see details in Section 3.1.), as observed. In this antibody concentration interval, no significant effect on the diameter size of adalimumab‐loaded PLGA NPs was observed, with particle size values ranging from 280 ± 11.4 nm in all formulations (*α* >0.05). This was in good agreement with results previously obtained by Xie et al.^[^ ^27^ ^]^ who reported no statistically significant differences over the PLGA NPs size prepared by the double‐emulsion/solvent evaporation solvent technique due to an increase on the antibody amount, in NPs.

### 4.3 Stability Studies

#### 4.3.1 Stability to Storage

**Figure 5:**
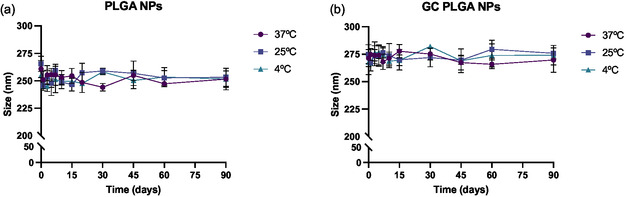
Resulting data of the stability‐to‐storage study for PLGA‐based NPs, where size and PDI changes were studied at three different temperature conditions. Graph a) displays the results for the ADA PLGA NPs while the graph b) shows the resulting data for the ADA–GC PLGA NPs.

**Figure** **5** shows the resulting data for the stability to storage study of adalimumab‐loaded PLGA NPs. As presented, drug‐loaded nanosystems did not suffer significantly apparent changes in terms of size, size distribution and surface charge, suggesting that these systems are stable over the studied period for the three tested temperature subsets. However, considering the characteristics of the drug itself, adalimumab, refrigerated storage is preferable to the other two options to maximize the drug stability overtime.

#### 4.3.2 Stability to pH

**Figure 6:**
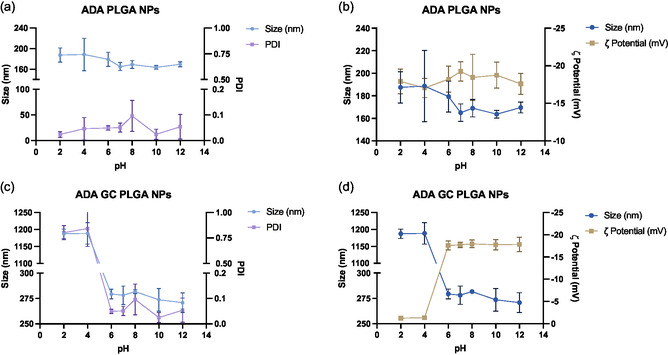
Resulting data of the stability‐to‐pH study. a,c) Graphs show the size and PDI changes over the studied pH interval for ADA PLGA NPs and ADA–GC PLGA NPs, respectively, while b,d) the graphs display the size and *ζ* potential variations along the same pH interval for the same type of formulations.

**Figure** **6** shows the resulting data for the stability to pH study of adalimumab‐loaded PLGA NPs. As observed, NP's size remained unchanged along the studied pH interval, being supported by minimal changes in both polydispersion and surface charge. This suggests that the resulting systems are robust and remain stable to changes in the pH of the medium.

#### 4.3.3 Stability to Ionic Strength

**Figure 7:**
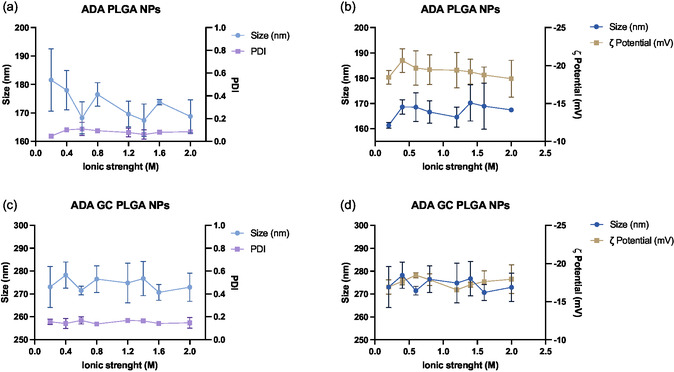
Resulting data of the stability‐to‐ionic strength study. a,c) Graphs show the size and PDI changes over the studied ionic strength interval for ADA PLGA NPs and ADA–GC PLGA NPs, respectively, while b,d) the graphs display the size and *ζ* potential variations along the same ionic strength interval for the same type of formulations.

**Figure** **7** shows the resulting data for the stability to ionic strength study of adalimumab‐loaded PLGA NPs. As presented, NP's size remained unchanged along the studied ionic strength interval, being supported by minimal changes in both polydispersion and surface charge. This is in good agreement with the stability‐to‐pH results, reinforcing the idea that the nanosystems are robust and remain stable to changes in the ionic strength of the medium.

### 4.4 DSC/TGA Analysis

**Figure 8:**
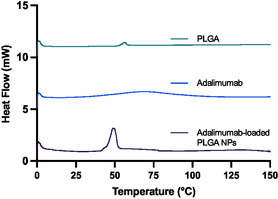
Differential scanning calorimetry (DSC) analysis of adalimumab‐loaded PLGA‐based NPs.

DSC curves of adalimumab‐loaded PLGA NPs and their main components are shown in **Figure** **8**. As can be seen from the DSC curves of the pure components, the glass transition temperature of PLGA (*T*_g_) was observed at 56.8 °C, indicating the amorphous nature of the polymer. The Tg value is in agreement with the literature, where PLGA polymers with a lactic acid to glycolic acid of 50:50 have been reported to have a Tg in the range of 30–40 °C.^[^ ^28^ ^]^ In contrast, DSC curve of pure adalimumab showed no changes in the range of temperature studied, indicating their amorphous nature.

The DSC curve of the adalimumab‐loaded PLGA NPs exhibited an exotherm peak at 48.2 °C. In addition to that, no changes in the weight were detected by TGA so the main peak could be attributed to the crystallization of the trehalose used as cryoprotector during the freeze‐drying process. No significant shifts or additional peaks were detected in the DSC curve of the formulations. This suggests minimal changes in the solid structure of adalimumab and the PLGA matrix, implying that the antibody is likely encapsulated within the polymer matrix without altering its thermal properties.

The TGA curve of the adalimumab‐loaded PLGA NPs did not display any peaks in the studied interval (0–150 °C), indicating that no degradation process has been observed.

### 4.5 FTIR Analysis

FTIR is a very sensitive assay for the analysis of the chemical surface of PLGA‐based NPs. FTIR spectra were employed to recognize functional groups in the resulting formulations, compared the standard spectra, proving that FTIR analysis is convenient for examining colloidal suspensions.

**Figure 9:**
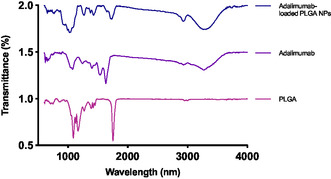
FTIR analysis of the adalimumab‐loaded PLGA‐based NPs.

First, a broad scan of the PLGA‐based NPs was performed from 400 to 4000 cm^−1^, as presented in **Figure** **9**. Among the various peaks present in the spectrum, several are already clearly identifiable as belonging to the functional molecules. The list of known wavenumber ranges obtained from FTIR databases.^[^ ^29^ ^]^

The absorption bands in the 1500–1600 cm^−1^ interval were presumably allocated to ionosorbed dioxygen species, whose obtention are associated with temperature increase from low temperature ranges.^[^ ^30^, ^31^, ^32^ ^]^ Different types of OH groups and their associated bending vibrations absorbed in the 1400–1900 cm^−1^ region, where their multiplicity may be associated with the different coordination states along the polymeric surface.^[^ ^33^ ^]^ At the end of the heat treatment, the absorption bands in the 3000–4000 cm^−1^ range may be related to the stretching OH vibrations of hydroxyl groups bonded to surface polymeric atoms.^[^ ^33^ ^]^

The FTIR spectrum of the pure PLGA polymer exhibited characteristic absorption bands at 2995 cm^−1^ (CH_3_ stretching), 2945 cm^−1^ (CH_2_ stretching), 1750 cm^−1^ (C=O stretching), and 1180 cm^−1^ (C—O—C stretching). These absorption bands are consistent with the reported values for PLGA polymers.

In contrast, the FTIR spectrum of adalimumab displayed characteristic absorption bands at 1650 cm^−1^ (amide I), 1550 cm^−1^ (amide II), and 1240 cm^−1^ (amide III). These absorption bands are indicative of the protein structure and are consistent with the reported values for adalimumab.

Likewise, the FTIR spectrum of the adalimumab‐loaded PLGA NPs showed a combination of the characteristic absorption bands of both PLGA and adalimumab. The absorption bands at 2995 cm^−1^ (CH_3_ stretching), 2945 cm^−1^ (CH_2_ stretching), and 1750 cm^−1^ (C=O stretching) are attributed to the PLGA polymer matrix, while the absorption bands at 1650 cm^−1^ (amide I), 1550 cm^−1^ (amide II), and 1240 cm^−1^ (amide III) are indicative of the presence of adalimumab within the NPs.

The FTIR spectrum of the adalimumab‐loaded PLGA NPs did not exhibit any significant shifts or changes in the absorption bands compared to the pure components. This suggests that there are no strong molecular interactions between the PLGA polymer and adalimumab, indicating that the antibody is likely encapsulated within the polymer matrix without undergoing significant conformational changes.

The presence of the characteristic absorption bands of both PLGA and adalimumab in the FTIR spectrum of the final formulation indicates that the structural integrity of both the polymer and the antibody is maintained. This is crucial for the potential application of the formulation in drug delivery systems, as it ensures that the antibody retains its biological activity. In conclusion, the FTIR spectroscopy analysis of PLGA NPs loaded with adalimumab provides evidence for the successful encapsulation of the antibody within the PLGA polymer matrix. The absence of significant molecular interactions and the maintenance of the structural integrity of both the polymer and the antibody suggest that the formulation is suitable for further studies.

### 4.6 X‐Ray Diffractometry Analysis

WAX diffraction analysis was conducted on the adalimumab‐loaded PLGA NPs, compared to the diffractograms of the pure components, to investigate the crystalline structure and molecular arrangement within the formulation.

**Figure 10:**
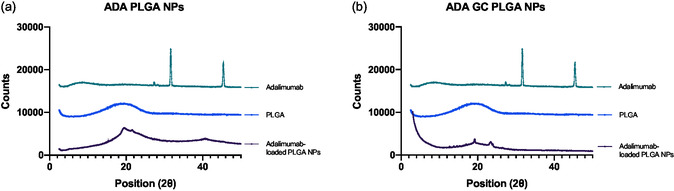
X‐ray diffraction analysis of the adalimumab‐loaded PLGA‐based NPs. a) Graph displays the results for the ADA PLGA NPs, while b) the graph shows the resulting data for the ADA–GC PLGA NPs.

The WAX diffractograms (WAXD) patterns of the adalimumab‐loaded PLGA NPs, pure PLGA polymer, and adalimumab are presented in **Figure** **10**. The WAXD pattern of the pure PLGA polymer did not show any diffraction peaks in the 2*θ* studied interval, corresponding to the amorphous nature of the polymer. This pattern is consistent with the reported diffraction angles for PLGA polymers with a lactic acid to glycolic acid ratio of 50:50.

In contrast, the WAXD pattern of adalimumab exhibited characteristic diffraction peaks at 2*θ* values of 23.8, 26.6, and 43.1°, corresponding to the semicrystalline nature of the protein. These peaks are consistent with the reported diffraction angles in previous studies.^[^ ^34^ ^]^

The WAXD pattern of the PLGA NPs loaded with adalimumab displayed diffraction peaks corresponding to a shift in the nature structure of the PLGA polymer. The peaks at 2θ values of 19.8 and 21.6° were observed. This suggests that the encapsulation of adalimumab minimally alter the amorphous structure of the PLGA matrix.

The presence of diffraction peaks corresponding to the antibody in the WAXD pattern of the adalimumab‐loaded NPs indicates that the molecular arrangement of the polymer apparently changes in the presence of the antibody. This suggests that adalimumab is likely concentrated within the core of the NPs.

The shift of the characteristic diffraction peaks of the components in the WAXD pattern of the formulation suggests that the antibody is successfully incorporated into the polymeric core. This is essential for the mechanical properties and stability of the NPs in drug delivery applications.

### 4.7 In Vitro Release Study

**Figure 11:**
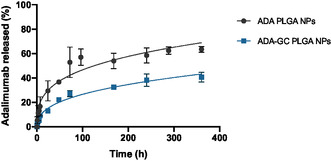
In vitro release study of adalimumab‐loaded PLGA‐based NPs.

The assessment of the in vitro release behavior of adalimumab‐loaded PLGA NPs was carried out based on a dialysis method. The release profile of adalimumab from the PLGA NPs exhibited a sustained and controlled pattern over the study period, as seen in **Figure** **11**. The cumulative release of adalimumab increased gradually with time, indicating a diffusion‐controlled release mechanism from the NPs. This sustained release behavior is desirable for maintaining therapeutic levels of the antibody over an extended period.

The release kinetics of adalimumab from the PLGA NPs followed a non‐Fickian diffusion mechanism, suggesting a combination of drug diffusion and polymer degradation contributing to the release process. The cumulative release of adalimumab from the PLGA NPs reached almost a 60% and a 40% for ADA PLGA NPs and ADA–GC PLGA NPs respectively at the end of the study period, demonstrating the efficient encapsulation and controlled release of the antibody from the nanoparticulate system. Thus, based on the results of the release study, as well as all previous characterization studies, the ADA GC PLGA NPs were selected as the most appropriate NPs for further in vivo biodistribution studies (see details in Section 4.10.).

### 4.8 Cytotoxicity Analysis

#### 4.8.1 BCOP

**Figure 12:**
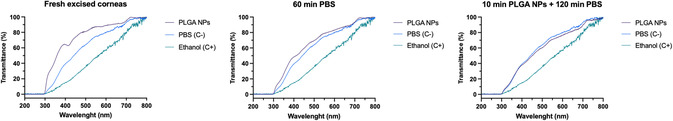
Evolution of the changes on the corneal transparency along the bovine corneal opacity and permeability (BCOP) study of adalimumab‐loaded PLGA‐based NPs.

The BCOP assay was conducted to evaluate the cytotoxic effects of PLGA NPs loaded with adalimumab on bovine corneal cells. The results showed that the NPs exhibited no corneal cytotoxicity (see **Figure** **12**). This indicates that the NPs were nontoxic to corneal cells at the concentrations tested. The assay also demonstrated that the NPs did not cause significant changes in corneal opacity or permeability, suggesting that they do not compromise the integrity of the corneal tissue.

#### 4.8.2 HET–CAM

**Figure 13:**
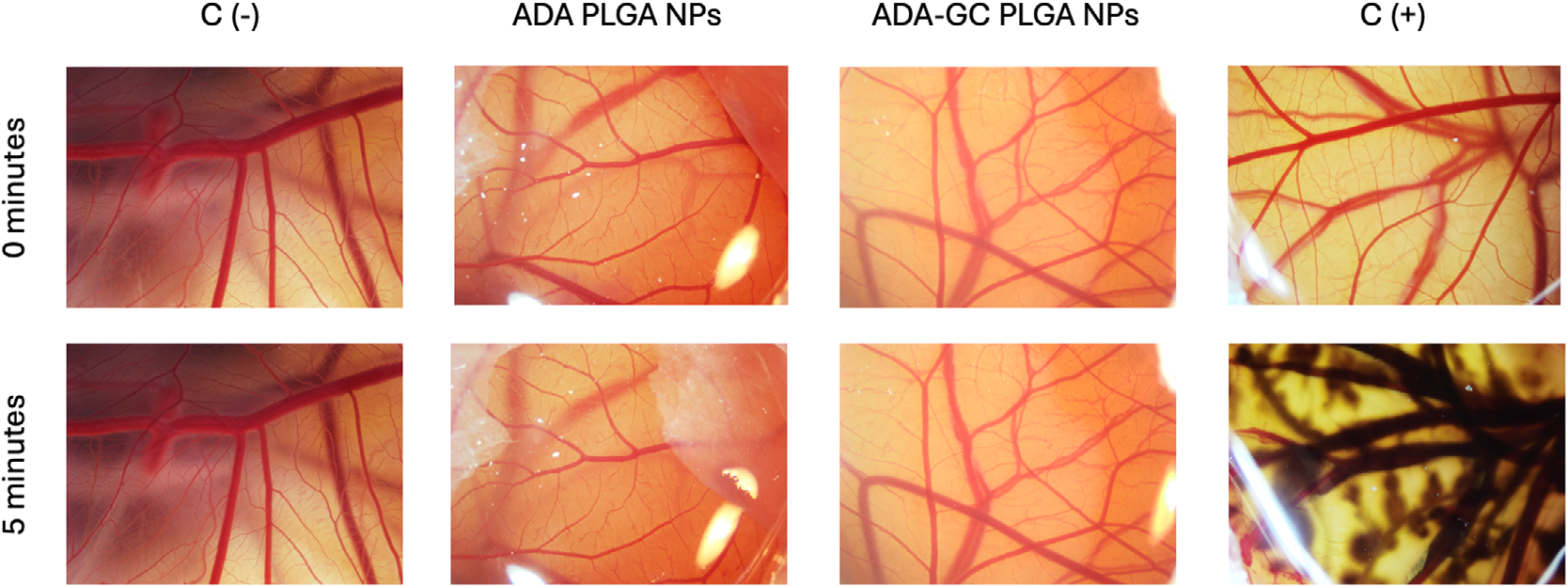
Hen's egg test on the chorioallantoic membrane (HET–CAM) study of adalimumab‐loaded PLGA‐based NPs.

The HET–CAM assay was used to assess the cytotoxic effects of adalimumab‐loaded PLGA NPs on the CAM of fertilized chicken eggs. The results indicated that the NPs exhibited no cytotoxicity (see **Figure** **13**). This suggests that the NPs may cause no damage to the CAM. Moreover, the assay also showed that the NPs did not induce any hemorrhage or necrosis phenomena, indicating that they do not cause severe tissue damage.

### 4.9 Biological Activity of Adalimumab‐Loaded PLGA NPs

The biological activity of adalimumab in PLGA NPs by an ELISA assay demonstrated robust viability of the encapsulated antibody. The ELISA analysis revealed a high viability of adalimumab within the PLGA NPs, with a viability rate exceeding 87 ± 4%. This indicates successful encapsulation and preservation of adalimumab's activity during the formulation process. Moreover, the release kinetics study exhibited sustained and controlled release of adalimumab from the PLGA NPs, further confirming the stability and viability of the encapsulated adalimumab.

The ELISA results also indicated that the bioactivity of adalimumab released from the PLGA NPs remained intact, showing comparable efficacy to free adalimumab. These findings suggest that the PLGA NP system effectively preserves the viability and bioactivity of adalimumab, making it a promising platform for the controlled and sustained delivery of this therapeutic agent.

### 4.10 PET Biodistribution and Pharmacokinetics of Intravitreal Adalimumab‐Loaded PLGA NPs

#### 4.10.1 Conjugation, Radiolabeling, and Quality Control

The conjugation between the N‐succinyl‐deferoxamine and the antibody showed an average ratio of 1.35 chelating groups per adalimumab molecule. In the case of N‐sucDf–adalimumab, 85% of the N‐sucDf groups attached to the antibody were available for ^89^Zr chelation. The conjugation process did not lead to adalimumab dimerization, thereby preserving the antibody's activity. The radiolabeling efficiency of ^89^Zr–adalimumab was 91.5%. Following an ultrafiltration process, the radiochemical purity of this radiolabeled compound was 99.69%. In the case of ^89^Zr–adalimumab‐loaded NPs, ultrafiltration was not performed based on the proof of concept previously carried out.

In this proof of concept, the determination of the radiotracer activity in both the supernatant and the sediment of both groups of NPs (blank and antibody loaded) showed completely opposite results: the radiotracer activity in the supernatant was 98.1 ± 0.2% in the blank NPs versus 2.4 ± 0.1% in the adalimumab‐loaded NPs, while in the sediment a radiotracer activity of 1.8 ± 0.1% was observed in the blank NPs versus 98.5 ± 0.2% in the antibody‐loaded NPs. This supports the idea that the incubation with the radiotracer was successful, and that the radiotracer was able to pass through the polymer matrix and become anchored to the antibody conjugate that was located on the core of the NPs.

#### 4.10.2 Intravitreal Pharmacokinetics

**Figure 14:**
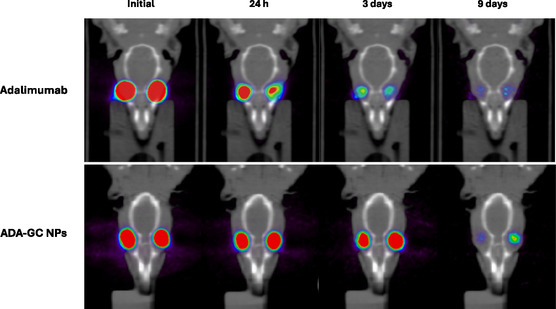
Coronal‐plane‐fused PET/CT images showing rat's head at different time points (initial, 24 h, 3 days, and 9 days) following intravitreal administration of ^89^Zr–adalimumab in both groups (adalimumab‐ and adalimumab‐loaded NPs). The color scale represents the radioactive uptake of ^89^Zr–adalimumab, ranging from lower (blue) to higher (red) intensity.

**Figure** **14** illustrates the radioactive uptake of ^89^Zr–adalimumab overtime as observed through PET/CT images in both the adalimumab and adalimumab‐loaded NPs groups. The data reveal a decline in uptake within the eye in both groups as the study progresses.

**Figure 15:**
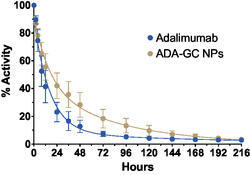
Percentage of remaining radioactivity in the eye overtime after intravitreal injection of ^89^Zr–adalimumab (*n* = 6 eyes) and ^89^Zr–labeled NPs (n = 4 eyes), *α* < 0.05 between 24 and 120 h.

The results of the elimination kinetics of adalimumab and ADA–GC NPs from the eye after intravitreal injection is shown in **Figure** **15**.

Both adalimumab and NPs exhibited a biexponential decrease in activity overtime. A two‐way ANOVA analysis, including time and formulation as variables, showed statistically significant differences in the percentage of remaining activity in the eye between 8 and 96 h for adalimumab and ADA–GC NPs. The results indicate lower eye clearance of adalimumab from NPs compared with free antibody. **Table** **3** presents the pharmacokinetic parameters obtained after fitting the pharmacokinetic profiles to a two‐compartment model developed in our previous work.^[^ ^21^ ^]^

**Table 3:** Intravitreal pharmacokinetic parameters of adalimumab‐ and adalimumab‐loaded GC NPs.

Parameter	Adalimumab		ADA–GC NPs	
	Mean	SD	Mean	SD
a [h^−1^]	0.0041	0.0200	0.0834	0.0378
b [h^−1^]	0.0566	0.0506	0.0121	0.0013
k_12_ [h^−1^]	0.0525	0.0300	0.0459	0.0108
k_21_ [h^−1^]	0.0044	0.0257	0.0219	0.0044
k_20_ [h^−1^]	0.0038	0.0116	0.0276	0.0238
S_*y*.*x*_	0.1141	0.0287	0.0587	0.0186
Root Mean Square Error (RMSE)	0.1041	0.0257	0.0520	0.0165
Akaike Information Criterion (AICc)	−58.07	6.1447	−74.1675	9.470

The fitting parameters suggest a good adjustment of the pharmacokinetic profiles to the two‐compartment model. The NPs notably exhibited a slower b constant with values slightly higher than the a constant. These values suggest that NPs were more rapidly distributed into the posterior chamber of the eye than free adalimumab, but with a slower clearance phase. Therefore, the results showed a modification in the distribution and elimination of adalimumab when injected as NPs.

#### 4.10.3 Blood Pharmacokinetics

**Figure 16:**
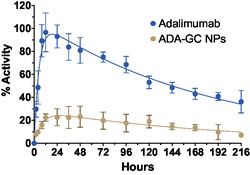
Percentage of blood radioactivity overtime after intravitreal injection of ^89^Zr–adalimumab in both rat groups (adalimumab (*n* = 6) and adalimumab‐loaded GC NPs (*n* = 4)), *α* < 0.05 at all‐time points, statistical analysis: two‐way ANOVA analysis.

**Figure** **16** shows the pharmacokinetic profiles of adalimumab and the adalimumab‐loaded GC NPs in blood after the intravitreal administration. An absorption phase was clearly observed in both profiles.

A clear difference in blood pharmacokinetic profiles was observed after the nanoencapsulation of adalimumab. The two‐way ANOVA analysis showed significant differences at all tested times. As shown in **Table** **4**, adalimumab‐loaded GC NPs showed that the *C*_max_ and the $AUC_{0}^{\infty }$ values were clearly lower compared to free adalimumab, suggesting that antibodies have a higher clearance from the blood when administered immobilized in NPs.

**Table 4:** Noncompartmental analysis of the blood pharmacokinetic profiles of adalimumab‐ and adalimumab‐loaded GC NPs.

Parameter	Adalimumab		ADA–GC NPs		Ratio
	Mean	SD	Mean	SD	
k [h^−1^]	0.005	0.0004	0.005	0.0006	0.93
t_1/2_ [h]	128.77	10.54	139.09	17.93	1.08
$AUC_{0}^{\infty }$ [% activity h]	19953.57	3346.86	5003.11	1345.67	0.25
C_max_ [% activity]	96.61	16.88	24.96	6.74	0.26

It is well known the recycling role of the FcRn receptor of the vascular endothelial cells, which contributes to the persistence of mAbs in blood, resulting in reduced clearance and increased half‐life of IgGs and mAbs in vivo. FcRn has been shown to be involved in the transcytosis of IgGs and mAbs across the epithelium. FcRn binds to the antibody, forming a complex that protects mAbs from degradation and promotes a recycling pathway to the vascular epithelium surface, whose pH (pH 7.4) facilitates the dissociation and release of mAbs into the circulation, thereby increasing their half‐life. In contrast, adalimumab loaded into GC NPs is unable to interact with FcRn receptors, inhibiting the recycling mechanism. Consequently, the NPs are quickly removed from the blood, improving their blood clearance.

#### 4.10.4 Whole‐Body Biodistribution

**Figure 17:**
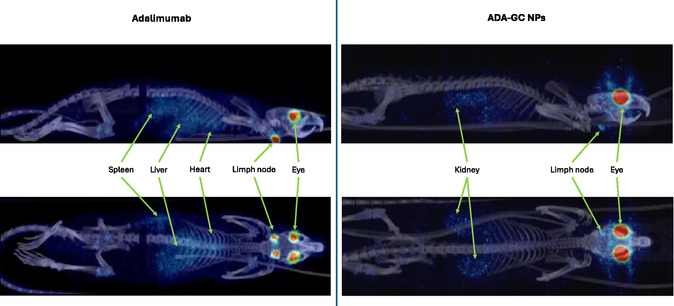
Rat whole‐body PET/CT images displayed in coronal (up) and sagittal (down) plane for ^89^Zr–adalimumab (left) and ^89^Zr–adalimumab‐loaded GC NPs (right), representing the antibody distribution in the organs with the highest uptake 3 days after intravitreal injection. The color scale represents the radioactive uptake of ^89^Zr–adalimumab, ranging from lower (blue) to higher (red) intensity.

As it can be seen in **Figure** **17**, the systemic distribution of free ^89^Zr–adalimumab and ^89^Zr–adalimumab‐loaded GC NPs can be observed in the whole rat body. In addition, two videos showing this whole‐body distribution can be found in Supporting Information.

**Figure 18:**
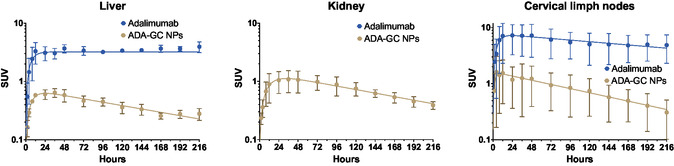
Activity (SUV) in the different organs (liver, kidney, and cervical lymph nodes) overtime after intravitreal injection of ^89^Zr–adalimumab in both rat groups (adalimumab‐ and adalimumab‐loaded GC NPs).

The antibody distribution in the organs (liver, kidneys, and cervical lymph nodes) was analyzed in the same way as blood data. A first‐order absorption one‐compartment model was the best pharmacokinetics fit for these results as it is shown in **Figure** **18**.

In all the organs where the uptake is observed, the adalimumab distribution completely changed when incorporated into NPs. The free antibody was distributed more rapidly and in greater proportion to the liver and lymph nodes, with almost no distribution to the kidneys. In contrast, the adalimumab‐loaded GC NPs were distributed to a lesser degree to the liver and lymph nodes, but there was a marked distribution to the kidneys.

These results are consistent with previous findings in healthy rats and rats with LPS‐induced uveitis.^[^ ^21^ ^]^ However, a clear change in behavior was observed with the adalimumab‐loaded GC NPs. The results showed a significant reduction in the distribution of adalimumab‐loaded GC NPs to the cervical lymph nodes (86% of reduction in ${\text{AUC}}_{0}^{216}$) and liver (88% of reduction in ${\text{AUC}}_{0}^{216}$). In contrast, significant concentrations of NPs were observed in the kidney, without detecting free adalimumab in this organ.

It is known that the most common mechanisms for eliminating mAbs from the blood are via the reticuloendothelial system and proteolysis in the liver. Under normal conditions, mAbs are too large to be filtered by the nephrons in the kidneys, so they are neither eliminated in the urine nor accumulated in this organ. Our results indicate that adalimumab‐loaded GC NPs can evade the reticuloendothelial system and liver, reducing accumulation in these organs. In addition to that, unlike free mAbs, adalimumab‐loaded GC NPs promote distribution and accumulation into the kidney. The adalimumab‐loaded GC NPs may be a good tool to reduce the immunogenicity of the antibody caused by the high permanence in blood and organism. This longer blood permanence does not help the antibody to enter the eye again, so it will not favor ocular bioavailability.

Kumar et al.^[^ ^35^ ^]^ studied the distribution of different NPs in mouse organs, finding that polymeric NPs are preferentially distributed in the liver (14.1%ID g^−1^) and spleen (9.4%ID g^−1^), followed by the kidney (2.7%ID g^−1^). In our case, a similar accumulation was observed in the lymph nodes and the kidney, but with less accumulation in the liver. The accumulation of PLGA NPs in the kidney has already been described by Deng et al.^[^ ^36^ ^]^ They localized particles of about 400 nm in the proximal tubular cells and showed the highest fluorescence intensity in the kidney among all other organs. NPs larger than 100 nm can enter the kidneys through exocytosis from epithelial cells of the peritubular capillary into the proximal tubular.^[^ ^37^ ^]^

#### 4.10.5 Autoradiography

**Figure 19:**
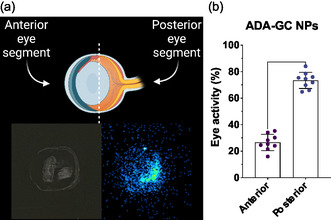
a) Autoradiography image of ^89^Zr–adalimumab‐loaded GC NPs eye distribution 9 days after intravitreal injection (right) and the same histological section (left). b) Percentage of ocular activity after 9 days of ^89^Zr–adalimumab GC NPs distribution in both anterior and posterior ocular segments.

Semiquantitative analysis of autoradiography images showing the distribution of adalimumab‐loaded GC NPs confirmed a higher uptake of ^89^Zr–adalimumab‐emitted activity in the posterior segment of the eye, suggesting the deposition and accumulation in the structures closest to the injection site (retina). In contrast, as it can be seen in **Figure** **19**, a significantly lower distribution was observed into the anterior segment, resulting in 26.58 ± 0.9% in the anterior segment and 73.41 ± 6.09% in the posterior segment in the studied slices.

Based on the literature,^[^ ^38^, ^39^ ^]^ intravitreally injected antibodies are distributed by diffusion to the anterior chamber of the eye, where they are removed into the systemic circulation by the trabecular and uveoscleral outflow. However, in the case of our NPs, a modification in the adalimumab distribution in the eye was observed with the accumulation of the radioactivity in the posterior segment of the eye. These autoradiographic results confirm those previously obtained in the ocular pharmacokinetic study.

## 5 Conclusion

In this comprehensive study on the design, development, and characterization of adalimumab‐loaded PLGA NPs, the potential of this innovative drug delivery system for enhancing the therapeutic efficacy of adalimumab, a monoclonal antibody targeting TNF‐*α*, was successfully demonstrated. The findings from this research shed light on the promising applications of PLGA NPs in the realm of controlled drug delivery and personalized medicine.

The optimization of formulation parameters resulted in the production of PLGA NPs with desirable characteristics, including a uniform size distribution, high drug loading capacity, and sustained release kinetics. These NPs exhibited excellent stability and biocompatibility, essential attributes for their potential clinical translation.

The in vivo studies demonstrated the potential of these NPs to enhance the therapeutic efficacy of adalimumab by improving the residence time of the adalimumab in the vitreous and promoting the distribution in the retina compared with free adalimumab. Surprisingly, NPs significantly modify the antibody distribution in blood and tissues, with accumulation in the kidney and less distribution to the liver and the spleen compared to free antibody. These changes suggest a modification in the clearance mechanism of the antibody from the vitreous when incorporated into the NPs.

## Conflict of Interest

The authors declare no conflict of interest.

## Author Contributions

**Xurxo García‐Otero**: data curation (equal); formal analysis (equal); investigation (equal); methodology (equal); software (equal); writing—original draft (equal); and writing—review and editing (equal). **Rubén Varela‐Fernández**: data curation (equal); formal analysis (equal); investigation (equal); methodology (equal); visualization (equal); writing—original draft (equal); and writing—review and editing (equal). **Andrea Cuartero‐Martínez**: data curation (equal); methodology (equal); and software (equal). **Noemí Gómez‐Lado**: data curation (equal); investigation (equal); methodology (supporting); software (supporting); and validation (supporting). **Miguel González‐Barcia**: formal analysis (supporting); funding acquisition (supporting); project administration (supporting); supervision (supporting); and writing—review and editing (supporting). **Cristina Mondelo‐García**: conceptualization (supporting); formal analysis (supporting); funding acquisition (supporting); resources (supporting); supervision (supporting); and visualization (supporting). **Carolina Feitosa**: formal analysis (supporting) and methodology (supporting). **Pablo Aguiar**: conceptualization (equal); formal analysis (equal); funding acquisition (equal); investigation (equal); methodology (equal); supervision (equal); and writing—review and editing (equal). **Anxo Fernández‐Ferreiro**: conceptualization (equal); funding acquisition (equal); resources (equal); supervision (equal); and writing—review and editing (equal). **Francisco J. Otero‐Espinar**: conceptualization (lead); formal analysis (lead); funding acquisition (lead); methodology (lead); project administration (lead); resources (lead); supervision (lead); validation (lead); writing—original draft (lead); and writing—review and editing (lead).

## Supporting information

Supplementary Material

## Data Availability

The data that support the findings of this study are available from the corresponding author upon reasonable request.
